# Supposed Case of Poisoning by Oxymuriate of Mercury

**Published:** 1827-10

**Authors:** John Frost


### POISONING.

*Supposed Case of Poisoning by Oxymuriate of Mercury.* By John Frost, F.A.S. F.L.S. &c.

On the 28th of July, I was requested by Mr. Cosgreave, the surgeon to the parish of St. Clement Danes, to visit a man of the name of Morgan, who had been brought from Lincoln's Inn-fields to the workhouse by a person, to whom he stated he had taken oxymuriate of mercury. He was found surrounded by a multitude, to whom he also stated that he had taken poison, and which rendered him an object of immediate attention. He appears formerly to have assisted in compounding medicines, and had purchased the corrosive sublimate under its technical appellation, which induced the vendor to sell it without a proper precaution. He stated he had purchased two drachms, which he had formed into pills; several of which he said he had swallowed; and, to prove the truth of his assertion, he gave eight or nine pills to the man who brought him to the workhouse, which he said, on being examined, would be found to contain the Hydrargyri Oxymurias.

On his admission, Mr. Cosgreave immediately emptied the stomach of its contents, by means of the stomach-pump, after which the stomach was alternately filled with warm water, and then emptied, until the water returned as clear as it was injected. A cathartic was then administered, and he was supplied with raw eggs and milk: in fact, he was treated as though he had actually taken corrosive sublimate.

Mr. Cosgreave requested me first to analyse the pills, which, as before mentioned, the patient stated would be found to contain oxymuriate of mercury. They were of a greyish aspect, not unlike the common bluepill. I triturated one with distilled water in a glass mortar, and then filtered the solution through bibulous paper, and treated the strained liquor with nitrate of silver, which threw down a copious, white, flocculent precipitate, which was entirely soluble in caustic ammonia, and which, of course, denoted the existence of muriatic acid. To another portion hydriodate of potassa was added, which precipitated the mercury of a yellow colour. Lime-water was then added to a fresh quantity of the filtrated solution, and threw down an orange-red precipitate in abundance. These tests very satisfactorily denoted the presence of a considerable portion of oxymuriate of mercury. In order still more to confirm these results, a drop of the filtrated solution was placed on a sovereign, and then placed in contact with the blade of a polished steel knife, when the mercury was immediately reduced in its metallic form on the surface of the gold.

Having satisfied Mr. Cosgreave and myself that the pills contained corrosive sublimate, I proceeded to the workhouse to visit the man, whom I found in a very emaciated condition, with an hectic countenance, his pulse weak, skin cool, and surface of the body pallid. He was very languid, and seemed to me to be suffering from exhaustion only, having no intense pain in the throat, or other symptoms attendant on the internal exhibition of any quantity of oxymuriate of mercury, which he said he had taken.

I submitted the contents of the stomach, as well as the alvine evacuations, to the tests for detecting the presence of mercury; and I conducted the experiments with the greatest precision, carefully diluting their contents in glass vessels with distilled water, in order to prevent the colouring matter from confusing the colour of the several precipitates. As there was no other evidence of his having taken corrosive sublimate than his own assertion, (which is supposed to have been made to the crowd which collected round him in the neighbourhood of Lincoln's Inn-fields, merely to excite their commiseration and charity,) and as he showed no decided symptoms of labouring under its action when I saw him, although he said that he had taken thirty grains only two days before, I took my departure, satisfied that he had not swallowed the poison, and having demonstrated satisfactorily to Mr. Cosgreave that there were no traces of corrosive muriate in any of the evacuations.

In the course of four days afterwards the man died, certainly of inanition, which was brought on by irregular habits and excessive indulgence in the use of fermented liquors. Mr. Cosgreave and myself examined the body after death. The only morbid appearances were adhesion of the liver to the diaphragm and peritoneum; the gall bladder was distended, and part of the left lobe of the liver indurated. The lungs partially adhered to the pleura. There was no marked inflammation of the œsophagus, stomach, or intestines.

I submitted the post-mortem contents of the stomach to the test of chemical analysis, and found no traces of any mineral poison having been in the stomach.

It appeared that, since the loss of his wife, he had been in a desponding way, although he was well provided for, being employed as a clerk in an attorney's office.

A person, calling himself a chemist, who attended the inquest, and who had given the deceased an emetic in the first instance, (which, by the bye, did not operate before the stomach-pump was used,) attempted to prove that the pills contained corrosive sublimate, by stating that *nitrate of silver* threw down a *yellow* precipitate with solutions of *corrosive sublimate*. This was certainly a new discovery to me, and I think it is the first time that such a coloured precipitate was ever obtained by nitrate of silver with solutions of oxymuriate of mercury; and, notwithstanding the evidence of Mr. Cosgreave, who made a conclusive experiment before the jury as to the detecting of the smallest quantity of corrosive sublimate, they nevertheless brought in a verdict that the deceased died by having taken poison.

*Bridge-street, Blackfriars; August 4th,* 1827.

